# An interview with Peter H. Buschang

**DOI:** 10.1590/2176-9451.19.6.026-036.int

**Published:** 2014

Dr. Peter Buschang is regent professor and director of orthodontic research. He has been at Texas A&M University Baylor College of Dentistry since 1988. Dr. Buschang received his PhD in 1980 from the University of Texas at Austin; he spent 3 years as a NIDR postdoctoral fellow at the University of Connecticut, and five years as a FRSQ scholar at the University of Montreal. Every year, Dr. Buschang teaches in 16 different courses, 7 of which he directs. In addition to more than 100 lecture hours per year, he spends hundreds of hours mentoring students. For his teaching efforts, Dr. Buschang was awarded the Robert E. Gaylord Award of Excellence in Orthodontic education in 1992, 1998, 2004, and 2010. He also gives 1-2 day evidence-based CE courses throughout the world. The residents he has taught recently honored him by pledging to fund the Peter H. Buschang Endowed Professorship of Orthodontics. His research interests pertain to craniofacial growth and assessment of treatment effects. Dr. Buschang has been funded regularly over the years by the Medical Research Council of Canada, *Fonds de le Recherche en Santé du Québec*, the NIH, and the American Association of Orthodontics Foundation. He has mentored over 140 Master's and PhD students, and 49 dental students. Dr. Buschang has published over 250 peer-reviewed articles, 15 book chapters and 198 abstracts. He has given over 150 invited talks and lectures in 14 different countries. For his work with the American Board of Orthodontics, Dr. Buschang was awarded the Earl E. and Wilma S. Shepard Award. Dr. Buschang is the only non-orthodontist ever to have been made an honorary member of both the American Association of Orthodontics (2005) and the Edward H. Angle Society of Orthodontics (2009), the two most prestigious orthodontic groups. 

Gerson Luiz Ulema Ribeiro and Helder Baldi Jacob

Dr. Buschang, you have recently published an article about Class I malocclusion and you have lectured extensively about its development. In your opinion, what should the orthodontist know in terms of etiology in order to prevent it from developing and improve the quality of treatment? (Gerson Luiz Ulema Ribeiro and Helder Baldi Jacob)

First and foremost, orthodontists should realize that most of the crowding that occurs post-treatment is due to the same factors that cause crowding in untreated individuals.^1^ Importantly, this caveat only applies if the orthodontist does not violate well established orthodontic principles (i.e. does not over-expand, does not excessively flare, ensures adequate retention, etc.). If you violate these principles, the teeth will move, usually within the first few months, and crowd. 

Teeth should be expected to crowd in approximately 50-60% of treated cases even when established principles have not been violated. The likelihood of crowding is even greater in untreated subjects. The basic problem is tooth movement. Tooth movements cause contact displacements, which is turn cause malalignment (Fig 1). For example, bite forces and large restorations produce an anterior component of force that can cause teeth to slip their contacts. The vertical eruption of teeth associated with growth, especially in hyperdivergent subjects, also causes teeth to move and slip contacts. Tooth movements can also be caused by tooth loss and abnormal emergence patterns. For example, the likelihood of crowding is greater when the first premolars emerge before the canines. Once contacts slip, the teeth involved will further move and/or rotate, causing other contacts to slip. The risk of slippage is greatly enhanced in individuals with point-to-point contacts and narrow arch forms. Orthodontists can prevent/minimize tooth movements by broadening interproximal contacts (this is especially important between canines and lateral incisors), informing patients that they must continue retention until growth has stopped (early to late 20's for females and males, respectively), and informing their referring dentists about restorations. 

**Figure 1.**
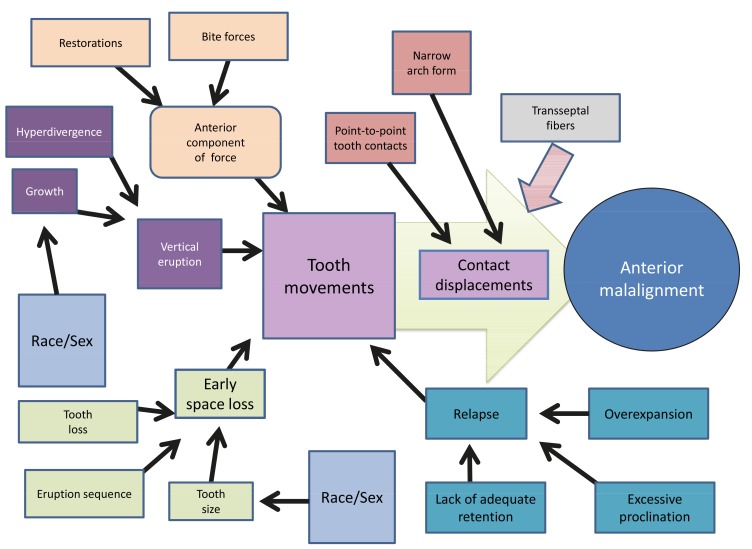
of the various factors that move teeth, change contacts, and lead to malalignment of the anterior dentition.

It has been shown that malocclusion is a recent event. Since the time span has been insufficient for genetic changes to have occurred, genotype must be adapting to environmental factors. What environmental factors might be expected to produce a hyperdivergent retrognathic phenotype? (Helder Baldi Jacob)

The hyperdivergent phenotype is primarily due to habitual lowered tongue and mandibular posture. Open-mouth posture alters the biomechanical environment and causes numerous adaptive responses, including supraeruption of maxillary teeth, narrow maxilla, often with crossbite, increased anterior face height, and open-bites, more posteriorly directed condylar growth leading to increased gonial angulation, long and narrow symphysis, and lower incisor retroclination (Fig 2). The two environmental factors most closely linked to open-mouth posture are weak muscles and compromised airways.^2^ From a historical perspective, reduced masticatory muscle forces best explain the increased prevalence of hyperdivergence, associated with a secular trend from prognathism to retrognathism. For example, Finns from the 16^th^ and 17^th^ centuries exhibited much less hyperdivergence than present day Finns, which has been attributed to softer present-day diets. A number of studies have shown a direct relationship between hyperdivergent growth tendencies and weaker masticatory muscle strength. Various studies have also shown that animals fed on softer diets (i.e. reduced masticatory stress) show many of the same morphological changes exhibited by hyperdivergent patients with weak muscles. Finally, and perhaps most convincingly, patients with muscular dystrophy and spinal muscular atrophy - both due to autosomal recessive genes that target the muscles - become progressively weaker and more hyperdivergent over time. For some individuals, strengthening the masticatory muscles may provide a way to reverse the development of the hyperdivergent phenotype. 

**Figure 2.**
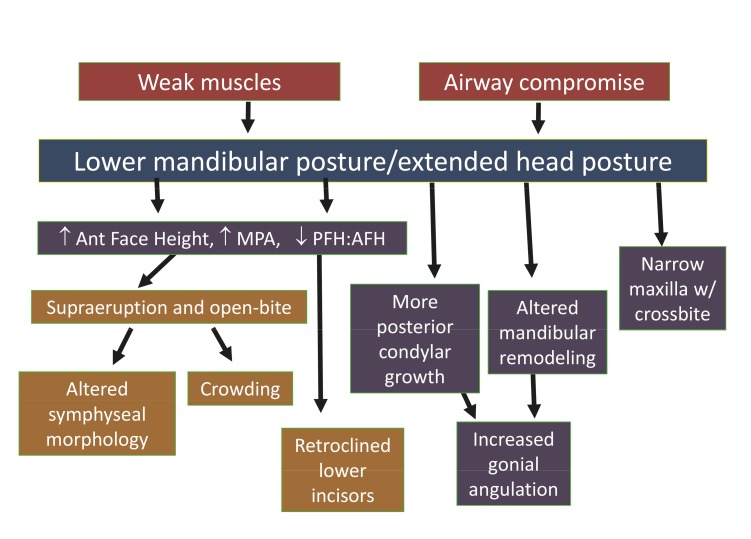
Chart of the development of the hyperdivergent retrognathic phenotype.

Chronic airway interferences have also been linked to development changes leading to the hyperdivergent phenotype. While compromised airways have in the past been difficult to objectively measure, there are simply too many studies associating long-term airway problems and hyperdivergence. There has to be a link. Harvold's classic experiments showed that when you block the nasal airway of primates, or force them to posture the mandible inferiorly, they develop steeper mandibular planes and larger gonial angles. Clinically, the relationship has been best established in patients with enlarged adenoids, probably because it is easier to measure upper than lower airway. Chronically enlarged tonsils have also been linked to hyperdivergence. More recently, children with sleep apnea and chronic allergic rhinitis have been shown to develop the same hyperdivergent, retrognathic, phenotype.

Craniofacial growth has been your research specialty during your entire life. What are the most important lessons you have learned and how do they contribute to treatment of malocclusions? (Luiz Gonzaga Gandini Júnior and Eustáquio Araújo)

Over the years, I have become more and more convinced that the orthodontist's understanding of craniofacial growth is essential for successfully treating patients. Understanding how, when and why untreated growth changes take place makes it possible to plan more effective treatments. As previously indicated in question #1 above, growth is probably the single most important determinant of crowding in both treated and untreated individuals. Orthodontists must understand that, in most cases, it is not treatment that causes the crowding that occurs after retention. Patients need to be retained according to their growth potential. Understanding growth, and the dental compensations associated with growth, can also make treatment more efficient.

Growth is also very important for treatment of Class II malocclusion. In most subjects, growth accounts for the majority of molar correction that normally occurs. The orthodontist simply has to prevent maxillary teeth from migrating forward, and allow mandibular growth to help with correction. This approach is particularly effective in the late mixed dentition phase, when leeway space is still available. In order to distinguish between growth and treatment effects, the orthodontist must be able to accurately superimpose radiographs. The ability to superimpose is one of the most important techniques that residents can learn during their training programs. In combination with a sound understanding of craniofacial growth and development, superimpositions provide the only way to distinguish between treatment and growth effects. Only after the orthodontist is able to make that distinction is he/she able to clearly identify the aspects of treatment that worked and did not work. Once you identify what did not work, corrective actions can be taken. One of the best ways to improve treatments is being able to control growth effects. 

For years, many orthodontists were taught that the mandible grows upward and backward, bringing the chin downward and forward. You have recently cast doubt on this relationship. In your opinion, what do orthodontists have to know in order to better understand chin projection? (Helder Baldi Jacob)

We have a series of publications showing that the most important determinant of chin projection is true mandibular rotation. For the longest time, orthodontists have wrongly focused on condylar growth as the major determinant of chin projection. For example, we have shown that the posterior displacement of the glenoid fossa of untreated subjects during growth is greater than the posterior growth of the condyle.^3^ This means that - all other things being equal - the chin should be displaced posteriorly. In fact, the chin of these subjects was displaced anteriorly (Fig 3). True rotation is the only explanation for this phenomenon. Once you come to this realization, it becomes clear why most traditional treatment approaches for hyperdivergent retrognathic Class II patients do not produce the desired skeletal effects.^4^ They often produce detrimental skeletal effects because they do not control rotation. To control rotation, you need to control the vertical eruption of teeth. This notion is based on the literature showing that greater rotation occurs during childhood than adolescence. The greatest rates of true rotation occur during the transition from the primary to mixed dentition.^5^ More recently, we showed that the true rotation that occurs during this transition is primarily due to the anterior space that is created with the loss of deciduous incisors.^6^ We have used this information to develop treatments for growing hyperdivergent Class II patients.^7^

**Figure 3.**
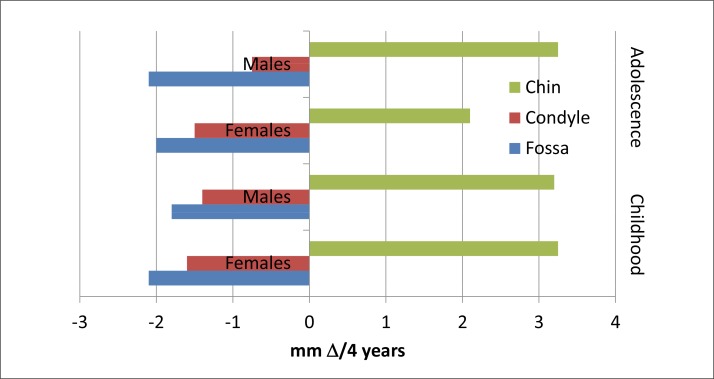
Posterior condylar growth, posterior fossa displacement and anterior chin displacement

Bionator therapy has been used to treat Class II malocclusion. It is a device that uses the propulsion of the mandible to achieve the necessary growth modifications. What are your views about the effects of this appliance and the fact that most of its effect is dentoalveolar and not skeletal, as desired? (Ary dos Santos-Pinto)

It is very difficult to perform orthopedics when you apply the forces to the teeth. This has been the basic dilemma of orthodontics for many years. The teeth are simply more plastic (move or change faster) than the bones. As such, it is not surprising that the major effects of the Bionator, and most other functional appliances, are dentoalveolar. However, there are predictable skeletal effects produced with Bionators. Functional appliances displace the mandible downward and forward, and redirect condylar growth backward. In doing so, the functional appliance produces a slightly larger mandible. It is larger because the distance between the condyle and chin increases more than expected without treatment. However, the orthodontist must realize that this size increase is limited and it is not due to increased amounts of condylar growth. Despite earlier claims to the contrary, functional appliances do not increase the growth of the condyles. The mandible adapts to its altered position by redirecting condylar growth more posteriorly. Redirection of condylar growth can be beneficial for hypodivergent Class II patients whose skeletal discrepancy is primarily due to a retrognathic mandible, rather than a prognathic maxilla. 

Recently you showed that Class II malocclusion was due to a difference in mandibular growth. Do you think that the Herbst appliance is the best therapeutic approach to the problem? Do you think that the fact that the appliance delivers an intrusive force toward upper molars is an advantage for patients with vertical growth patterns? (Ary dos Santos-Pinto)

As I have already indicated, I believe that functional appliances are appropriate for hypodivergent Class II patients because they have essentially the same mandibular growth potential as Class I's. However, the orthodontist must also consider the potential negative sequelae (i.e. downward and backward rotation of the mandible) that can be produced with functional appliances. These sequelae must be minimized for the appliance to be effective. As such, I do not think that the Herbst, or any other functional appliance, should be used for subjects with vertical growth patterns. We showed that when the Herbst is used in hyperdivergent patients, the ANB correction is due to changes in the SNA angle, not the SNB angle.^4^ In contrast, ANB correction with the Herbst in hypodivergent cases is primarily due to changes in the SNB angle, which is appropriate because most Class II cases are due to retrognathic mandibles. 

With respect to the Herbst's intrusive force directed toward the upper molar, it is important to remember that it is not sufficient to control the vertical movements of maxillary teeth. While absolute or sometime simply relative intrusion of maxillary molars is appropriate for most patients, treatment also has to control lower teeth. If you only intrude the upper molars, the lower molars will compensate, often limiting or compromising mandibular treatment effect.

Several approaches have been used to treat hyperdivergent Class II patients, including masticatory exercises, posterior bite blocks, vertical chin cups, vertical high pull headgears, and modified Thurow extraoral appliances. Is inhibition of vertical maxillary growth the best approach to treat the problem? (Ary dos Santos-Pinto)

Hyperdivergent Class II patients cannot be treated successfully by focusing on the maxilla. True forward rotation of the mandible is required to address the skeletal needs of hyperdivergent patients. There are only two treatment approaches that address both jaws and produce consistent results. Over 35 years ago, Pearson^8^ showed the vertical pull chin-cup therapy works. He was able to reduce the mandibular plane angle an average of almost four degrees, which is truly remarkable. However, his patients had to wear the appliance at least 12 hours per day and treatment often extended over many years. We performed a study in 2000^9^ which convinced me that (1) chin-cups work by rotating the mandible, and (2) they produce a number of morphological adaptations that are expected based on our understanding of rotation. The problem is that patients do not like to wear vertical pull chin-cups for a variety of reasons. 

With the advent of skeletal anchorage, it is now possible to effectively and efficiently treat hyperdivergent patients by either intruding posterior teeth or by preventing their eruption.^7^ Whether the orthodontist has to intrude depends on patient's growth potential. The amount of rotation that occurs is entirely under the control of the orthodontist. The good news is that there is an association between the amount of forward rotation and other orthopedic effects. The greater the true rotation, the greater the chin projection, molar correction, reduction in anterior lower face height, reduction in mandibular plane angle, and increase in posterior height. This treatment can address most of hyperdivergent patient's problem! 

You have lectured and published about a novel approach to treat hyperdivergent retrognathic Class II patients using miniscrews. After analyzing the cases treated in your Department, what should the orthodontist be more effective in doing so as to treat this kind of patients? (Helder Baldi Jacob and Gerson Luiz Ulema Ribeiro)

We were funded by the NIDCR to determine whether intrusion of posterior segments of teeth produces mandibular rotation, chin advancement and improvements of lower facial profiles. We also needed to know if miniscrews remained stable and were well tolerated throughout treatment. It is important to emphasize that all patients were treated exactly the same. In the maxilla, they all had a Dentaurum Variety SP RPE, there were occlusal rests to control the eruption of second molar, and Sentalloy coil springs (150 grams) extending from 8 mm IMTEC:3M miniscrews that had been inserted bilaterally between first molars and second premolars (Fig 4A). 

**Figure 4.**
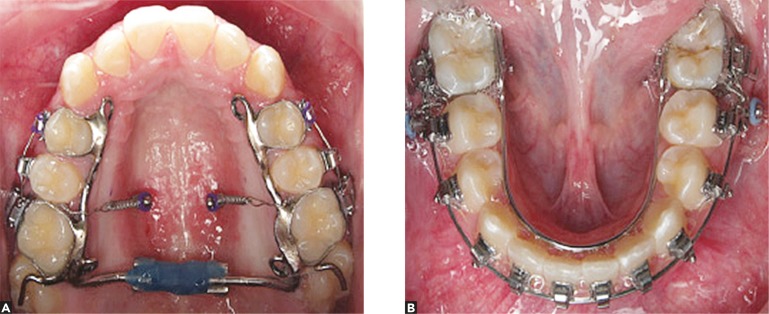
Maxillary (A) and mandibular (B) appliances used for treatment of Class II hyperdivergent growing patients.

The same miniscrews were placed between mandibular first molars and premolars. For most patients, an intrusive force was not needed in the mandible. The teeth were held in place with ligatures extending from the miniscrews to the 0.016 x 0.022-in SS arch wire on the teeth. In a couple of non-growing patients who needed intrusion, the same Sentalloy coil springs (150 grams) were used. The lingual arch was only used when teeth were being intruded. 

As indicated in the last question, the most important lesson that we learned from this study was that the treatment works, and has the potential to produce remarkable orthopedic effects - equivalent to those produced surgically. We also learned that the orthodontist can control treatment by monitoring outcomes. The orthodontist must take intraoral photographs every two months to evaluate whether or not positive treatment effects are occurring. If the patient is growing, you should see improvements in overbite (frontal photo) and in the molar/canine relationship (side view photos) within 2-4 months (Fig 5). If you do not see improvements, you need to increase the amount of upper intrusion, start holding the lower dentition, or maybe even start intruding the lower dentition.

**Figure 5.**
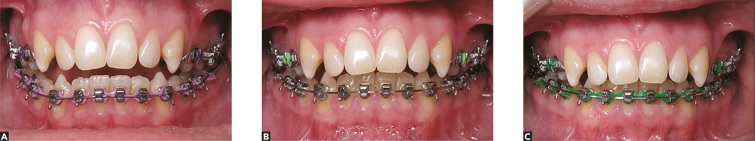
Class II hyperdivergent growing patient treated by means of miniscrews on the max

We also learned that the orthodontist has to take a progress cephalogram at the end of the orthopedic phase of treatment to determine whether he/she has attained sufficient orthopedic correction. More orthopedic correction is often necessary after the dental relationships have been corrected. We also learned that none of the patients reported miniscrews to be painful; they did find them to be somewhat uncomfortable, but less so than RPE. Finally, we found that miniscrews were remarkably stable. Our success rate was over 95%, which was due to the insertion techniques.^10^

## Class III malocclusion has been characterized in various ways. In your opinion, what is the main reason that leads a person to develop this type of malocclusion? (Gerson Luiz Ulema Ribeiro)

**Figure 6.**
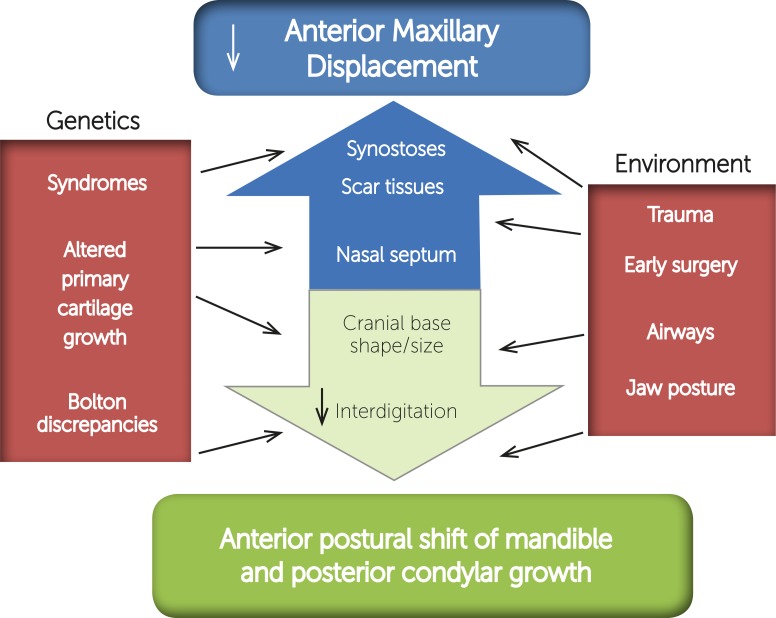
Chart showing genetics and environment influence in the development of Class III malocclusion.

I believe both genetics and environment are involved in the development of Class III (Fig 6). For those Class III patients whose problem is primarily maxillary retrusion, genetics could play a role in causing premature synostoses of maxillary sutures. Genetics could also reduce the growth of primary cartilage in the midface. Environmental factors can also limit the amount of anterior maxillary displacement that takes place, including cleft lip/palate surgery, and traumatic synostosis of the midface.

For most Class III's, the skeletal problem is primarily in the mandible. I believe genetics predisposes many of them to decreased interdigitation of posterior teeth, which allows the mandible to shift anteriorly. Numerous studies have shown that Class III's often have smaller cranial base angles. Genetics might be expected to play a role because synchondroses are well established growth centers. Tooth size is also highly heritable, so it is not surprising to learn that Class III's have Bolton discrepancies due to relatively larger mandibular teeth. There are also some environmental factors, such as jaw posture and airway, that could cause the mandible to be shifted anteriorly. Importantly, these factors all tend to disocclude the teeth (decrease interdigitation). Lack of interdigitation explains why Class III's have less stable occlusal relationship (i.e., smaller posterior occlusal contact and near contact areas) than Class II and Class I malocclusions.^11^

I do not believe that most Class III patients exhibit excessive amounts of condylar growth. They do have larger mandibles, when measured from the condyle to the chin, due to more posteriorly directed condylar growth. As previously described for hyperdivergent Class II's, condylar growth of Class III's is directed more posterior because the mandible is displaced down and forward. The literature comparing Class III's and Class I's rarely shows significant differences in ramus height; and Björk's Class I and Class III cases, whose mandibles were superimposed on metallic implants, show essentially the same amount of condylar growth. This suggests that the longer mandibles that characterize Class III's are primarily due to positional rather than genetic factors.

What are the pros and cons of miniscrew implants compared to miniplates in Class III correction? On which basis should the clinician choose one over the other? Can these approaches be used similarly in the mixed dentition? (Eustáquio Araújo and Luiz Gonzaga Gandini Júnior)

Both miniscrew implants (MSI's) and miniplates are capable of providing the skeletal anchorage needed to treat Class III patients. At the moment, miniplates offer greater stability than MSI's,^12^ which makes sense because the plates connect two miniscrews and provide greater primary stability.^13^ However, the failure rates of MSI's have been improving over the years. In 2008, most practicing orthodontists reported that MSI's fail less than 25% of the time.^14^ A systematic review of MSI's, performed in 2009 with 27 studies, reported a failure rate of 16.4%, which was more than two times greater than the failure rates of miniplates (7.3%). More recently, Papageorgiou et al^15^ systematically assessed 19 studies pertaining to 4987 MSI's placed in 2,281 patients and reported an overall failure rate of 13.5%. Further declines in failure rates of MSI's might be expected to occur. We recently showed that it is possible to have less than 5% of MSI's failure following a rigorous insertion protocol.^10^ The disadvantages of miniplates pertain to placement site locations and costs. The plates and screws are expensive and two surgeries are required for placement and removal. Due to potential damage to the teeth, miniplates are usually not placed in primary or mixed dentition patients, and placement sites are limited to the proximity of the roots. MSI's are less expensive and can be placed in more locations. We have developed a treatment protocol that uses MSI's to anchor the teeth and SAIF springs to protract the maxilla and control the AP displacement of the mandible. There is also work being done using longer and somewhat larger miniscrews that require only one surgical intervention.

During the AAO in Philadelphia you showed some Class III malocclusions treated by means of MSI's in the mixed dentition. How does this approach differ from the use of rapid expansion of the maxilla combined with facial masks? (Gerson Luiz Ulema Ribeiro)

Miniscrews are used to indirectly anchor the teeth. SAIF springs transmit 150 gram/side to a transpalatal bar in the maxilla and the mandibular arch wires (in the canine area). These forces serve to protract the maxilla and retract the mandible. Importantly, we have found that there is substantially less force transmitted to the miniscrews when using indirect anchorage. Our approach differs from the face mask in two important ways. First, greater orthopedic and less orthodontic movements might be expected. The orthopedics are greater because tooth movements are limited by MSI's. Facemasks produce substantially greater dental movements. Secondly, the mandible is not rotated downward and backwards, as with the facemask. The line of force is much higher than with the facemask. It is directed posterior/superiorly rather than posterior/inferiorly. It prevents the chin from coming forward and redirects condylar growth in a more superior/anterior direction, which causes the gonial and mandibular plane angles to decrease. The force system produces effects similar to the system used by De Clerck and coworkers.^16^

Over the last few years, you have conducted research assessing tooth movement accelerators. I would like to know whether you believe this topic will evolve over time and whether you really believe this procedure will become a routine in orthodontics offices. (Luiz Gonzaga Gandini Júnior)

I absolutely believe that orthodontists will be able to routinely move teeth much faster in the future. But this depends entirely on future research. We and others have been working hard trying to better understand how corticotomies affect bone biology.^17^ There is strong experimental evidence that corticotomies speed up tooth movements because the surgical insult produces a Regional Acceleratory Phenomenon (RAP) effect. RAP reduces the amount and density of bone that the tooth has to be moved through.^18^^,^ 19 ^,^ 20 Multiple studies show that it approximately doubles the rate of movement. There is moderately strong evidence that the amount of injury matters. With greater injury you get more tooth movement^21^ because there is more osteopenia.^22^ There is weak and somewhat contradictory evidence for the other, less invasive, approaches (i.e. those that do not require flap surgeries). For example, there is a clinical trial indicating that three micro-perforations 2-3 deep increase the rates of canine movements.^23^ However, a split mouth (25, 2-mm deep, holes drilled without flaps on one side) experimental dog study showed no differences in tooth after three months. We produced 60 awl injuries 2-3 mm deep on the buccal and lingual surfaces around premolars, which did not accelerate tooth movements because the osteopenia associated with the injuries was limited to the cortical bone.^24^ Finally there is limited evidence that piezocision accelerates tooth movements.^25^

Importantly, the RAP effect and tooth movement rate increases are of relatively short duration. They only lasted for 1-2 months in the experimental animals that have been evaluated. Assuming that bone turnover rates of dogs are 1.5 greater than in humans, this suggests that the effects of corticotomies should be limited to 2-3 months in humans, during which time 4-6 mm (i.e., twice the normal amount per month) of tooth movement might be expected. Unfortunately, clinical trials validating these notions are limited. However, there was a split-mouth design showing that the rate of canine retraction with corticotomies was also approximately doubled, and that there were no differences in tooth movements between sides after 3 months.^26^
